# Morphogenesis and development of midgut symbiotic organ of the stinkbug *Plautia stali* (Hemiptera: Pentatomidae)

**DOI:** 10.1186/s40851-019-0134-2

**Published:** 2019-05-31

**Authors:** Sayumi Oishi, Minoru Moriyama, Ryuichi Koga, Takema Fukatsu

**Affiliations:** 1 Department of Biological Sciences, Graduate School of Science, The University of Tokyo, Tokyo, 113-0033 Japan; 2 Bioproduction Research Institute, National Institute of Advanced Industrial Science and Technology (AIST), Tsukuba, 305-8566 Japan; 3 Computational Bio Big Data Open Innovation Laboratory (CBBD-OIL), National Institute of Advanced Industrial Science and Technology (AIST), Tsukuba, 305-8566 Japan; 4 Graduate School of Life and Environmental Sciences, University of Tsukuba, Tsukuba, 305-8572 Japan

**Keywords:** Stinkbug, *Plautia stali*, Gut, Symbiotic bacteria, *Pantoea*, Symbiotic organ, Midgut crypt, Development, Evolution

## Abstract

Diverse insects are intimately associated with microbial symbionts, which play a variety of biological roles in their adaptation to and survival in the natural environment. Such insects often possess specialized organs for hosting the microbial symbionts. What developmental processes and mechanisms underlie the formation of the host organs for microbial symbiosis is of fundamental biological interest but poorly understood.

Here we investigate the morphogenesis of the midgut symbiotic organ and the process of symbiont colonization therein during the developmental course of the stinkbug *Plautia stali*. Upon hatching, the midgut is a simple and smooth tube. Subsequently, symbiont colonization to the posterior midgut occurs, and thickening and folding of the midgut epithelium proceed during the first instar period. By the second instar, rudimentary crypts have formed, and their inner cavities are colonized by the symbiotic bacteria. From the second instar to the fourth instar, while the alimentary tract grows and the posterior midgut is established as the symbiotic organ with numerous crypts, the anterior midgut and the posterior midgut are structurally and functionally isolated by a strong constriction in the middle. By the early fifth instar, the midgut symbiotic organ attains the maximal length, but toward the mid fifth instar, the basal region of each crypt starts to constrict and narrow, which deforms the midgut symbiotic organ as a whole into a shorter, thicker and twisted shape. By the late fifth instar to adulthood, the crypts are constricted off, by which the symbiotic bacteria are confined in the crypt cavities and isolated from the midgut main tract, and concurrently, the strong midgut constriction in the middle becomes loose and open, by which the food flow from the anterior midgut to the posterior midgut recovers.

This study provides the most detailed and comprehensive descriptions ever reported on the morphogenesis of the symbiotic organ and the process of symbiont colonization in an obligatory insect-bacterium gut symbiotic system. Considering that *P. stali* is recently emerging as a useful model system for experimentally studying the intimate insect-microbe gut symbiosis, the knowledge obtained in this study establishes the foundation for the further development of this research field.

## Introduction

Diverse insects and other organisms are associated with microbial symbionts, which play substantial roles in their adaptation to and survival in the natural environment. The beneficial roles of the microbial symbionts encompass assistance of food digestion [1, 2], provisioning of essential nutrients [3, 4], facilitated resistance to natural enemies [5, 6], improved tolerance to environmental stresses [7, 8], degradation of noxious chemicals [9, 10], promotion of plant adaptation [11, 12], and many others. Hence, understanding of host mechanisms that underpin the establishment and maintenance of the microbial symbiosis is of fundamental biological interest.

Some microbial symbionts are endocellular like *Buchnera* in aphids, where the symbiotic bacteria are harbored in specialized cells and organs for symbiosis, called the bacteriocytes and the bacteriomes, respectively [3, 13]. Meanwhile, other microbial symbionts, such as *Pantoea* or *Burkholderia*, are extracellular in a variety of stinkbugs, in which the symbiotic bacteria are harbored in the inner cavity of a specialized region of the alimentary tract, so-called the midgut symbiotic organ [14, 15].

Many plant-sucking stinkbugs are associated with symbiotic bacteria of beneficial nature in a specialized region of the posterior midgut, where numerous sac-like structures, called crypts, harbor the bacteria within the inner cavity extracellularly [14–18]. The midgut of the stinkbugs is generally differentiated into structurally distinct regions, among which the posterior end region is specialized for microbial symbiosis [19–21], but the developmental and functional aspects of the midgut regions have been poorly understood. In these stinkbugs, upon oviposition, adult females secrete a symbiont-containing excrement from the anus, and either smear it onto the eggshell, place it beside the eggs, or cover the eggs with a large amount of the excrement. Upon hatching, newborn nymphs immediately ingest the excrement and orally acquire the symbiont, thereby establishing the vertical symbiont transmission [22–28]. Symbiont removal either by sterilizing the egg surface or removing the symbiont-containing excrement results in growth retardation, elevated mortality and/or sterility of the insect host [25, 26, 29–38], highlighting the fatal importance of the gut symbiotic system in stinkbugs.

What developmental processes and mechanisms underlie the formation of the insect cells and organs specialized for microbial symbiosis has been poorly understood and remains as an unanswered issue in the evolutionary developmental biology. While notable old histological descriptions, some of which were comprehensive but most of which were fragmentary snapshots, may be available (reviewed in [16]), recent detailed studies have been limited to only a few cases, which are represented by the pea aphid *Acyrthosiphon pisum* [39–41], the seed bug *Nysius plebeius* [42], and the bean bug *Riptortus pedestris* [43, 44].

Here, we focus on the brown-winged green stinkbug *Plautia stali* (Hemiptera: Pentatomidae), which has been known as a devastating pest of various fruits and crops [45] and recently emerging as a laboratory model for studying the insect-microbe gut symbiosis. *P. stali* is associated with a specific γ-proteobacterial symbiont, allied to *Pantoea* spp., within numerous crypts arranged in four rows along the posterior midgut [28, 29]. As a notorious agricultural pest, stable and reliable system for rearing *P. stali* in the laboratory has been established for decades [46], including the recent development of aseptic rearing procedures [47]. While an uncultivable gut symbiont (so-called symbiont A) is fixed in the mainland Japan, the gut symbiotic microbiota is distinct and polymorphic in southwestern islands of Japan, where an uncultivable gut symbiont (symbiont B) coexists with multiple cultivable gut symbionts (symbionts C–F) in the same islands and populations, which plausibly represents an ongoing process of symbiont diversification and/or replacement in natural insect populations [28]. For the cultivable symbionts, genetic and experimental manipulations are feasible, and for the host insect, knockdown of gene expression by RNA interference works efficiently [28, 47].

In this study, we investigated the morphogenesis of the midgut symbiotic organ and the process of symbiont colonization during the developmental course of *P. stali* in detail, by which we identified the first instar and the fifth instar as the important developmental stages for shaping the gut symbiotic system in the insect.

## Materials and methods

### Insect rearing

A mass-reared laboratory strain of *P. stali*, which had been established from adult insects collected in Tsukuba, Ibaraki, Japan, was used in this study. All populations of *P. stali* in the mainland Japan, including the Tsukuba population, are associated with a specific γ-proteobacterial gut symbiont, so-called symbiont A, which is phylogenetically close to *Pantoea dispersa* and allied environmental bacteria [28]. The symbiont A of *P. stali* is uncultivable, with a degenerate genome, and is essential for normal growth and survival of the host insect [28, 29, 47]. Ovipositing adult females of *P. stali* smear a symbiont-containing excrement onto the egg surface, and newborn nymphs actively suck it for around 30 min to acquire the symbiont immediately after hatching [28]. The insects maintained in mass-stock culture were reared on raw peanuts and water supplemented with 0.05% ascorbic acid (DWA) in large plastic containers at 25 ± 1 °C under a long-day regime of 16 h light and 8 h dark, as described previously [47]. It has been reported that raw peanut is the best laboratory food for *P. stali* in comparison with other seeds, grains, and fruits that were tested [46]. Egg masses were collected from the stock culture, their hatching was monitored every day, and newborn nymphs from them were reared and used for experiments. For the purpose of developmental monitoring, each newborn nymph was individually reared in a plastic Petri dish (90 mm in diameter, 20 mm in depth) with 3 raw peanuts and a cotton ball soaked with DWA, and inspected for molting or eclosion from 15:00 to 20:00 every day. For quantitative PCR (qPCR) and fluorescence in situ hybridization (FISH), about 10 insects were kept in each plastic Petri dish with 5–8 raw peanuts and a cotton ball soaked with DWA. The insects were monitored for molting or eclosion from 15:00–20:00 every day, and sorted according to the date of molting or eclosion for developmental staging. The rearing Petri dishes were kept in climatic chambers at 25 ± 1 °C under a long-day regime of 16 h light and 8 h dark. The food and DWA were renewed once a week.

### Insect dissection and sample preparation

The insects were dissected in Petri dishes filled with a phosphate-buffered saline (PBS: 137 mM NaCl, 8.1 mM Na_2_HPO_4_, 2.7 mM KCl, 1.5 mM KH_2_PO_4_ [pH 7.4]) using fine scissors, forceps, razors and/or needles, and photographed under a stereomicroscope (S8APO; Leica). For comparing the symbiont titers between the symbiotic regions and the non-symbiotic regions of the alimentary tract, adult insects 1 week after eclosion were dissected, and the dissected tissue samples were individually kept in a ultracold freezer at − 80 °C. For quantifying the symbiont population dynamics during the developmental course of *P. stali*, nymphs and adults of each developmental stage were collected 1 day after hatching, molting or eclosion. First-, second- and third-instar nymphs were preserved in acetone [48]. Fourth- and fifth-instar nymphs and adults were dissected in PBS and their symbiotic organs were kept in a ultracold freezer at − 80 °C. For analyzing the symbiont population dynamics after egg hatching, we performed strict developmental staging of the newborn nymphs of *P. stali*. We collected many egg masses, each of which consisted of around 14 eggs, and on the fifth day after oviposition, the eggs were inspected every hour to collect newborn nymphs. These strictly-staged newborn nymphs were preserved in acetone every 24 h for 5 days.

### DNA preparation and quantitative PCR (qPCR)

Each insect tissue sample was homogenized in a plastic tube with a lysis buffer (100 mM NaCl, 10 mM Tris-HCl [pH 8.0], 1 mM EDTA, 0.2% [w/v] SDS), extracted with phenol-chloroform-isoamylalcohol (25:24:1), precipitated and washed with ethanol, dried in air, and dissolved in a DNA suspension buffer (10 mM Tris-HCl [pH 8.0], 0.1 mM EDTA).

We quantified the symbiotic bacteria by qPCR in terms of bacterial *groEL* gene copies essentially as described previously [27]. An 80 bp region of *groEL* gene of the symbiont A was targeted by the primers AgroL1265F (5′-TTG CGG CGA AAA TCG CAG CT-3′) and AgroL1345R (5′-TCG CAC GCA GAG CAA CCT TA-3′). The PCR reaction mixture was composed of 375 nM each of the primers, 50 × ROX low, 2 × KAPA SYBR FAST qPCR Master Mix Universal (KAPA Biosystems), 5 μl of template DNA solution (1/20 of total DNA per insect), and water in a total of 20 μl. A standard curve was drawn using a 0.4 kb *groEL* gene segment as standard DNA samples, which was PCR-amplified with the primers AgroL1084F (5′-AAA GAG AAA CTG CAG GAG CG-3′) and AgroL1508R (5′-CGG GTC ACT TTG GTT GGA T-3′), purified and serially diluted. For each sample quantification, two sample replicates were measured with standard DNA samples containing 8.5 × 10^2^, 10^3^, 10^4^, 10^5^, 10^6^ and 10^7^ *groEL* gene copies, respectively.

### Histology and fluorescence in situ hybridization (FISH)

FISH was conducted using a fluorochrome-labeled oligonucleotide probe SymAC89R (5′-Alexa555-GCA AGC TCT TCT GTG CTG CC-3′) that targeted bacterial 16S rRNA of the symbiont A as described [49]. Dissected symbiotic organs were fixed in PBS containing 4% paraformaldehyde (PFA) for 3 h at room temperature, and washed with PBS containing 0.1% Tween 20 (PBST). For whole-mount FISH, the organs were washed and equilibrated with a hybridization buffer (20 mM Tris-HCl [pH 8.0], 0.9 M NaCl, 0.01% sodium dodecyl sulfate, 30% formamide) and then incubated in the hybridization buffer supplemented with 100 nM probe and 1 μg/ml 4′,6-diamidino-2-phenylindole (DAPI) overnight at room temperature in a dark box. After the incubation, the samples were washed with PBST, mounted with 80% glycerol or Slowfade Gold Antifade Mountant (Thermo Fisher), and observed under a fluorescence stereomicroscope (M165FC; Leica) or a laser confocal scanning microscope (LSM700; Zeiss). For FISH of tissue sections, after fixation with PFA, the samples were dehydrated and embedded by following the Technovit 8100 protocol (Heraeus Kulzer). Then, the embedded samples were cut into 2 μm sections on a microtome (RM2255; Leica), mounted on glass slides, and incubated with the hybridization buffer supplemented with 100 nM probe and 1 μg/ml DAPI for 2 h at room temperature in a humidified dark box. Then, the samples on the glass slides were washed with PBS, mounted with Slowfade Gold Antifade Mountant, and observed under an epifluorescence microscope (LSM700; Zeiss). In order to confirm the specificity of the fluorescent signals, we conducted no-probe control experiments, RNase-treated control experiments, and competitive suppression control experiments.

### Visualization of food passage

The experimental insects were provided with DWA containing 0.05% Brilliant Blue FCF as drinking water. For facilitating water uptake, we started the experiment using the insects that had been kept without drinking water for a day. After the treatment for 2 days, the insects were dissected, and their alimentary tracts were observed and photographed under a stereomicroscope (S8APO; Leica).

## Results

### Midgut symbiotic organ of *P. stali*

**Fig. 1:**
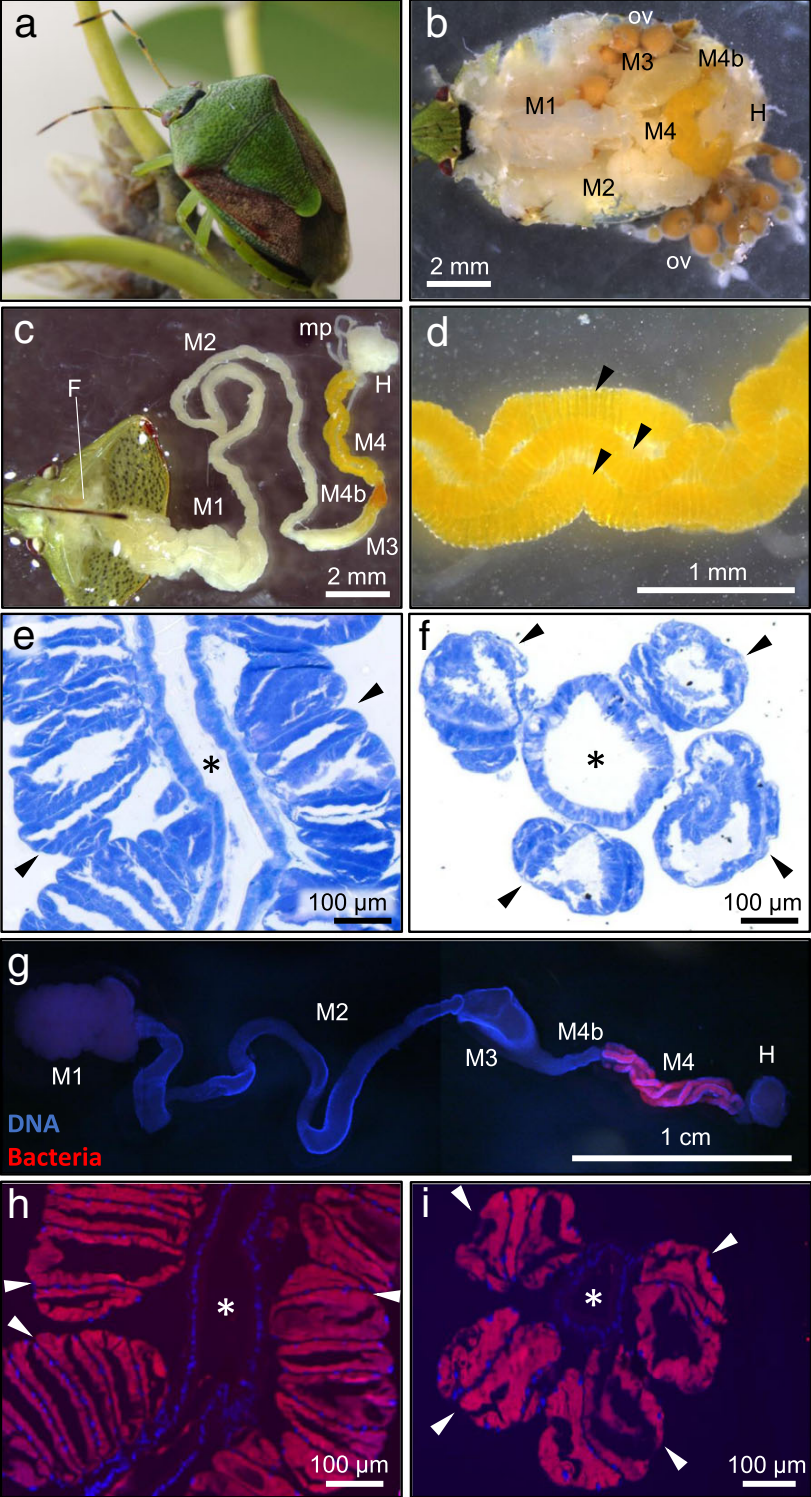
Symbiotic organ of *P. stali.* **a** An adult insect. **b** Dorsal view of a dissected insect. **c** An isolated alimentary tract, in which M4 represents the symbiotic organ. **d** An enlarged image of the symbiotic organ with numerous crypts in four rows. Three rows are seen (arrowheads), whereas another row is behind. **e**, **f** Toluidine blue staining of a longitudinal section (**e**) and a cross section (**f**) of the symbiotic organ. **g** FISH detection of the symbiotic bacteria in a dissected alimentary tract. **h**, **i** FISH detection of the symbiotic bacteria on a longitudinal section (**h**) and a cross section (**i**) of the symbiotic organ. In **g**–**i**, blue and red signals indicate host’s nuclear DNA and symbiotic bacteria, respectively. Arrowheads and asterisks show the crypt rows and the main tract of the midgut symbiotic organ, respectively. Abbreviations: F, foregut; M1, midgut first section; M2, midgut second section; M3, midgut third section; M4b, bulb-like section aboral to M4; M4, midgut fourth section, the symbiotic organ with crypts; H, hindgut; ov, ovary; mp, Malpighian tubule

We dissected adult insects of *P. stali* (Fig. 1a and b) to observe their midgut symbiotic organ. The alimentary tract was differentiated into a series of structurally distinct regions from oral to aboral as follows: foregut, a short tubular region directly connected to mouth; midgut first section (M1), a stomach-like enlarged region; midgut second section (M2), a long tubular region; midgut third section (M3), a moderately enlarged region; midgut bulb section (M4b), a small swollen region adorally connected to M4; midgut fourth section (M4), a yellow-colored twisted region with numerous crypts specialized for hosting the symbiotic bacteria; and hindgut, an end region of the alimentary tract to which Malpighian tubules connect (Fig. 1c). Microscopic observations revealed an elaborate structural configuration of the symbiotic organ, where numerous crypts are neatly arranged in four rows along the main tract of the midgut M4 region (Fig. 1d-f). FISH targeting bacterial 16S rRNA visualized the symbiotic bacteria localized to the inner cavity of the crypts in the midgut M4 region (Fig. 1g-i).

### Developmental stages of *P. stali*

**Fig. 2:**
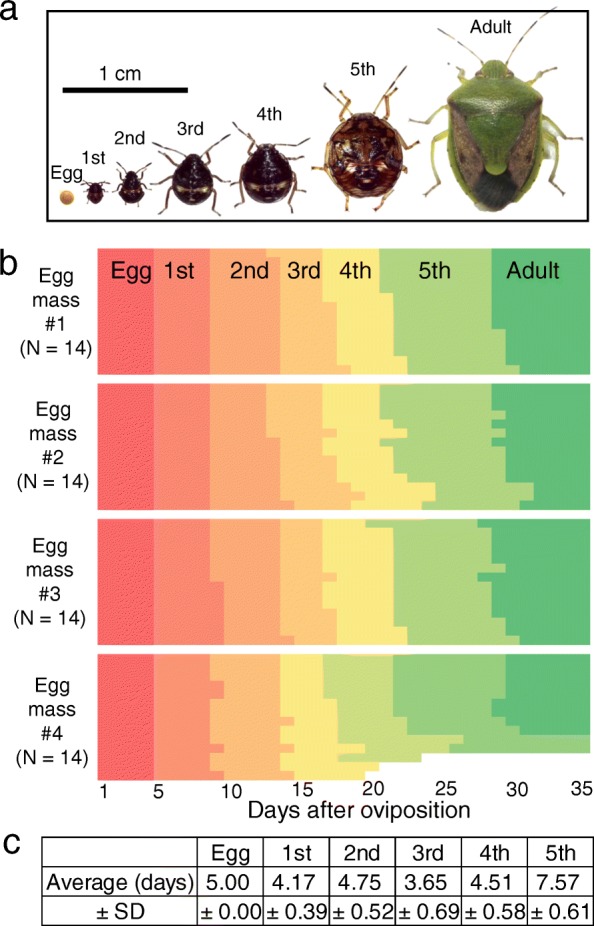
Developmental stages of *P. stali*. **a** Photos taken on the first day of each stage. **b** Developmental patterns of hatching, molting and emergence of 56 insects derived from four egg masses at 25 °C, under 16 h light and 8 h dark, and fed with raw peanuts and water supplemented with ascorbic acid. **c** Duration of the developmental stages of 51 insects (days, mean ± standard deviation). Note that 5 of 56 insects that we inspected either died as nymphs or failed to attain adulthood within 35 days, and thus are omitted from the analysis

In order to investigate the developmental process of the midgut symbiotic organ, we maintained *P. stali* under a defined rearing condition (25 °C, 16 h light and 8 h dark, fed with raw peanuts and water supplemented with 0.05% ascorbic acid). In general, the insects exhibited well-synchronized developmental patterns, around 5.0 days as eggs, 4.2 days as first instar nymphs, 4.8 days as second instar nymphs, 3.7 days as third instar nymphs, 4.5 days as fourth instar nymphs, and 7.6 days as fifth instar nymphs (Fig. 2a-c). After eclosion, adult insects survived up to 2 months, and reproductively-active young females laid around 14 eggs every one or 2 days.

### Population dynamics of symbiotic bacteria during development of *P. stali*

**Fig. 3:**
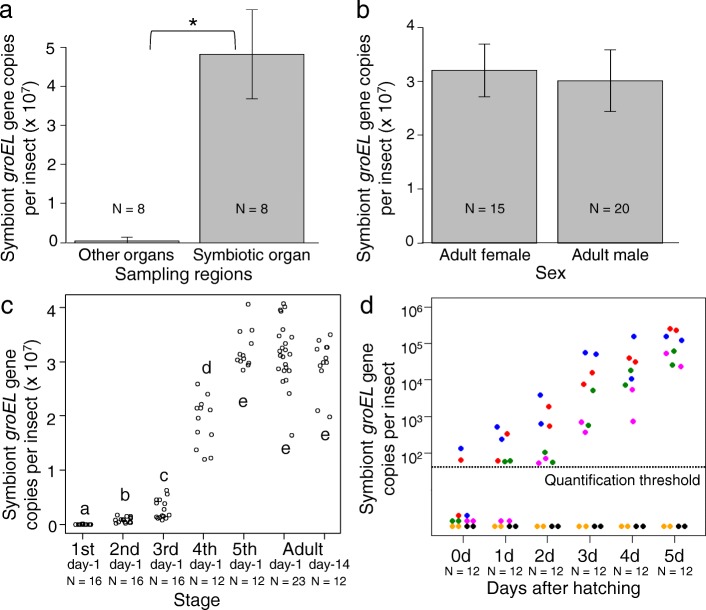
Population dynamics of the symbiotic bacteria in the developmental course of *P. stali*. Symbiont titers were evaluated in terms of symbiont *groEL* gene copies using qPCR. **a** Comparison of the symbiont titers between the midgut symbiotic organ (M4) and the other regions (M1, M2, M3, M4b and hindgut) of the alimentary tract. Columns and bars show means and standard deviations. Asterisk indicates statistically significant difference (Mann-Whitney U-test; *P* < 0.01). **b** Comparison of the symbiont titers between adult females and adult males. Columns and bars show means and standard deviations. The difference is statistically not significant (Man-Whitney U-test; *P* = 0.309). **c** Population dynamics of the symbiotic bacteria in the developmental course of *P. stali*. Each dot shows the symbiont titer in an insect. Different alphabetical letters (a-e) indicate statistically significant differences (Steel-Dwass test; *P* < 0.01). Abbreviations: 1st day-1, first instar nymphs 1 day after hatching; 2nd day-1, second instar nymphs 1 day after molting; 3rd day-1, third instar nymphs 1 day after molting; 4th day-1fourth instar nymphs 1 day after molting; 5th day-1, fifth instar nymphs 1 day after molting; Adult day-1, adults 1 day after eclosion; Adult day-14, adults 2 weeks after eclosion. **d** Population dynamics of the symbiotic bacteria in newborn nymphs of *P. stali*. Each dot shows the symbiont titer in an insect and each color indicates the insects derived from the same egg mass. Quantification threshold value of qPCR was set at 50 *groEL* gene copies per insect. Abbreviations: 0d, first instar nymphs on the day of hatching; 1d, first instar nymphs 1 day after hatching; 2d, first instar nymphs 2 days after hatching; 3d, first instar nymphs 3 days after hatching; 4d, second instar nymphs 4 days after hatching or on the day of molting; 5d, second instar nymphs 5 days after hatching or 1 day after molting. Note that logarithmic growth of the symbiotic bacteria was observed in the insects derived from the egg masses shown in red, blue, green and magenta, whereas little population growth of the symbiotic bacteria was observed in the insects derived from the egg masses shown in black and orange

We quantified the symbiotic bacteria by qPCR targeting bacterial *groEL* gene copies. When the alimentary tracts were dissected from adult insects, separated into the midgut M4 region (M4) and the other regions, and subjected to DNA extraction and qPCR, the symbiotic bacteria were exclusively detected in the midgut M4 region (Fig. 3a). When the symbiont titers were quantified and compared between adult females and males, similar levels of symbiont titers were detected with no statistically significant difference (Fig. 3b). In the developmental course of *P. stali*, the symbiont titers showed an exponential increase from the first instar to the fourth instar, and then reached a plateau at the fifth instar and on (Fig. 3c). When newborn nymphs were subjected to strict developmental staging (0, 1, 2, 3, 4 and 5 days after hatching) and symbiont quantification, in general, the symbiont titers increased steadily (Fig. 3d), reflecting the successful symbiont colonization, proliferation and establishment in the nymphal midgut. Meanwhile, we found that, in nymphs derived from two specific egg masses (shown in black and orange dots in Fig. 3d), the symbiont titers exhibited no increase, reflecting the failure of symbiont acquisition probably because of maternal failure in symbiont excretion and smearing onto the eggs.

### Development of midgut symbiotic organ and colonization process of symbiotic bacteria in *P. stali*

**Fig. 4:**
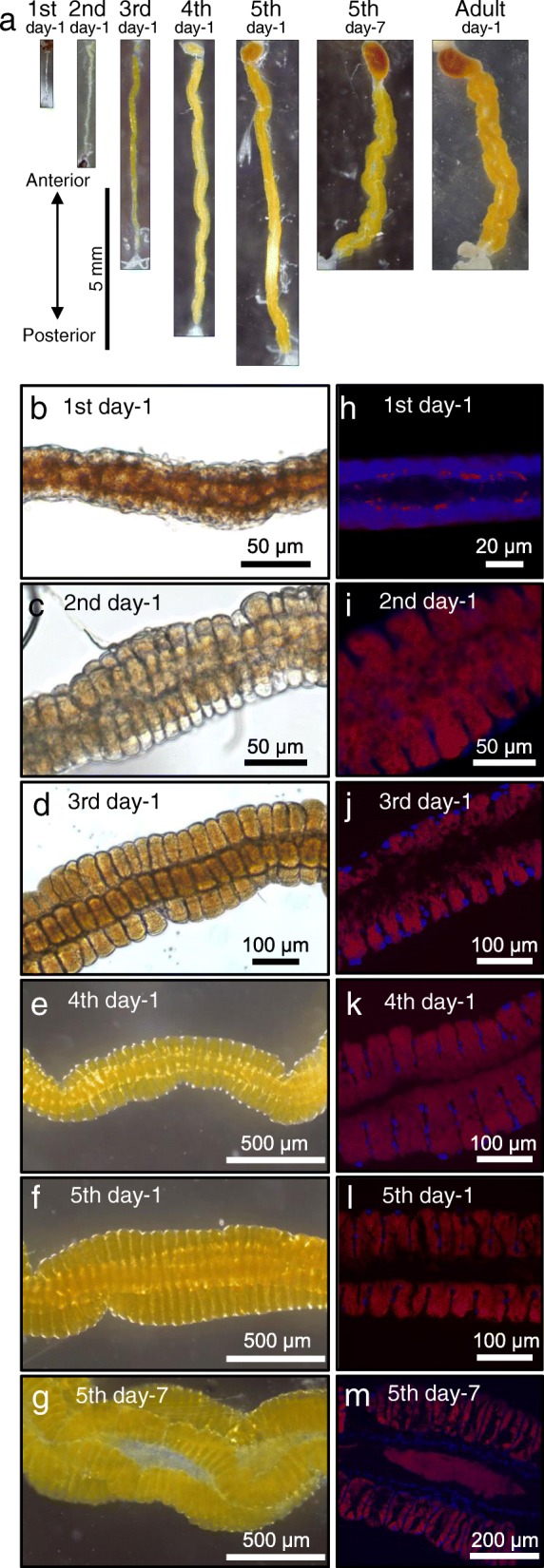
Development of the symbiotic organ and colonization process of the symbiotic bacteria in *P. stali*. **a** Dissected symbiotic organs at different developmental stages: 1st day-1, first instar nymph 1 day after hatching; 2nd day-1, second instar nymph 1 day after molting; 3rd day-1, third instar nymph 1 day after molting; 4th day-1, fourth instar nymph 1 day after molting; 5th day-1, fifth instar nymph 1 day after molting; 5th day-7, fifth instar nymph 7 days after molting; Adult day-1, adult insect 1 day after eclosion. **b-g** Enlarged bright-field images of the symbiotic midgut. Note that the crypts are obscure at the first instar and become evident at later stages. **h**–**l** FISH visualization of the symbiotic bacteria on longitudinal tissue sections of the midgut symbiotic organ. Note that the crypts are obscure and the symbiotic bacteria are sparse at the first instar, whereas the crypts are evidently formed and densely populated by the symbiotic bacteria at later stages. **b, h** First instar nymphs 1 day after hatching. **c**, **i** Second instar nymphs 1 day after molting. **d**, **j** Third instar nymphs 1 day after molting. **e**, **k** Fourth instar nymphs 1 day after molting. **f**, **l** Fifth instar nymphs 1 day after molting. **g** Fifth instar nymphs 7 days after molting. **m** Fifth instar nymph 7 days after molting. In **h**–**m**, blue and red signals indicate host’s nuclear DNA and symbiotic bacteria, respectively

We collected the following *P. stali* samples representing the defined developmental stages: first instar nymphs 1 day after hatching, second instar nymphs 1 day after molting, third instar nymphs 1 day after molting, fourth instar nymphs 1 day after molting, fifth instar nymphs 1 day after molting, fifth instar nymphs 7 days after molting, and adult insects 1 day after eclosion. When these insects were subjected to dissection of their alimentary tract and observation of their midgut symbiotic organ, an interesting pattern emerged. From first instar to fifth instar 1-day, the symbiotic midgut M4 region became longer as the host development proceeded. However, from fifth instar 1-day to adult, the symbiotic midgut M4 region became shorter instead, whereas the midgut region progressively thickened and twisted during the period (Fig. 4a). These observations suggest that some important morphogenetic events occur in the midgut symbiotic organ at the fifth instar stage.

The dissected alimentary tracts representing the defined developmental stages were subjected to morphological observations (Fig. 4b–g) and FISH visualization of the symbiotic bacteria (Fig. 4h-m). In the first instar nymphs, crypts were structurally not evident and symbiont signals were found only sporadically on the inner surface of the gut tube (Fig. 4b and h), which were in contrast to the well-developed crypts full of the symbiotic bacteria at the second to fifth instar stages (Fig. 4c–g and i-m). The midgut crypts arranged in four rows were already evident at the second instar stage (Fig. 4c). These observations suggested that crypt formation in the midgut M4 region must occur during the first instar period.

### Morphogenesis of midgut symbiotic organ at early developmental stages of *P. stali*

**Fig. 5:**
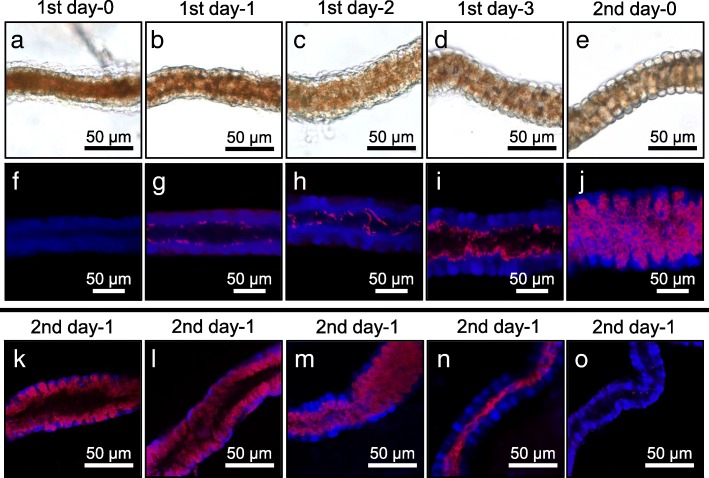
Morphogenesis of the symbiotic organ at early developmental stages of *P. stali*. **a–e** Bright-field images of the symbiotic organs. **f–j** Confocal microscopic images of the symbiotic organs, in which symbiotic bacteria are visualized by FISH in red and host’s nuclear DNA is counterstained in blue. **a**, **f** First instar nymph on the day of hatching. **b**, **g** First instar nymph 1 day after hatching. **c**, **h** First instar nymph 2 days after hatching. **d**, **i** First instar nymph 3 days after hatching. **e**, **j** Second instar nymph on the day of molting or 4 days after hatching. **k–o** Confocal microscopic images of the symbiotic organs of second instar nymphs 1 day after molting, where the levels of crypt formation and symbiont colonization exhibit considerable variation

Hence, we investigated the early development of the midgut symbiotic organ and the colonization process of the symbiotic bacteria in detail by sampling strictly-staged first and second instar nymphs of *P. stali*. In the first instar nymphs on the day of hatching, the posterior midgut was a simple smooth tube with few symbiont signals (Fig. 5a and f). In the first instar nymphs 1 day after hatching, the midgut epithelium became somewhat rough with sparse symbiont signals on the inner surface (Fig. 5b and g). In the first instar nymphs 2 days after hatching, the roughness of the midgut epithelium and the symbiont colonization proceeded (Fig. 5c and h). In the first instar nymphs 3 days after hatching, the midgut epithelium was thickened and folded, whose inner surface was densely colonized by the symbiotic bacteria (Fig. 5d and i). In the second instar nymphs on the day of molting, rudimentary crypts were formed and their inner cavities were densely populated by the symbiotic bacteria (Fig. 5e and j). These observations clarified the initial process of crypt formation and symbiont colonization in the midgut symbiotic organ, and confirmed that the process mainly occurs during the first instar period of *P. stali*. However, it should be noted that the levels of crypt formation and symbiont colonization may exhibit some variation among individuals (Fig. 5k-n). Though rarely, we occasionally came across *P. stali* nymphs whose midgut contained few symbiotic bacteria (Fig. 5o). Plausibly, these nymphs failed to acquire a sufficient number of symbiotic bacteria orally from the eggshell to establish the infection, which must be destined to suffer retarded growth and high mortality [28, 29, 47]. Interestingly, rough midgut epithelium with crypt-like structure was observed in these aposymbiotic nymphs (Fig. 5o). These observations suggested the possibility that the initial process of crypt formation may proceed autonomously even in the absence of the symbiotic bacteria. Alternatively, the initial process of crypt formation may be triggered not by the symbiotic bacteria but by the maternal secretion smeared on the egg surface that newborn nymphs ingested.

### Morphogenesis of midgut symbiotic organ at late developmental stages of *P. stali*

**Fig. 6:**
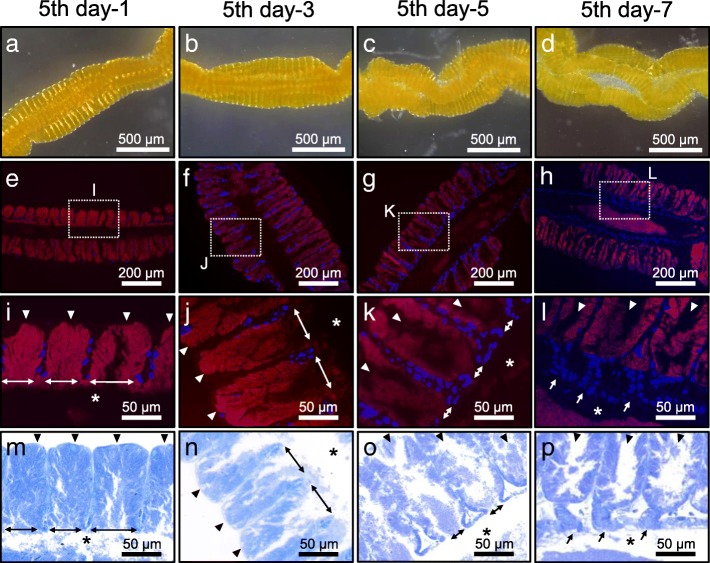
Morphogenesis of the symbiotic organ at late developmental stages of *P. stali*. **a–d** Bright-field images of the symbiotic organs. **e–h** FISH visualization of the symbiotic bacteria in red on tissue sections of the symbiotic organ, where host’s nuclear DNA is counterstained in blue. **i-l** Magnified images of (**e-h**). **m–p** Adjacent tissue sections to **i–l** stained with toluidine blue. **a**, **e**, **i**, **m** Fifth instar nymph 1 day after molting. **b**, **f**, **j**, **n** Fifth instar nymph 3 days after molting. **c**, **g**, **k**, **o** Fifth instar nymph 5 days after molting. **d**, **h**, **l**, **p** Fifth instar nymph 7 days after molting. In **i-p**, arrowheads highlight the crypts, asterisks show the main tract, two-way arrows indicate the crypt openings to the main tract, and arrows show the pedicel-like structures formed with the crypt closure

From the second instar to the fourth instar, the midgut symbiotic organ exhibited a simple growth, in which the organ size increased in accordance with the increased body size (see Fig. 4a). Next, therefore, we focused on the development of the midgut symbiotic organ and the process of symbiont colonization at the fifth instar stage of *P. stali*, because the midgut symbiotic organ exhibited characteristic shortening, thickening and twisting during the period (see Fig. 4a). In the fifth instar nymphs 1 day after molting, the symbiotic midgut region was straight in shape, in which each of numerous crypts was full of the symbiotic bacteria and open to the midgut main tract (Fig. 6a, e, i and m). In the fifth instar nymphs 3 days after molting, notably, the most basal region of each crypt started to narrow in width (Fig. 6b, f, j and n). In the fifth instar nymphs 5 days after molting, the basal region of each crypt was strongly constricted, almost separating each crypt cavity from the midgut main tract (Fig. 6c, g, k and o). In the fifth instar nymphs 7 days after molting, which were just prior to adult eclosion, each crypt cavity was structurally isolated from the midgut main tract, being connected to the main tract by a short pedicel-like epithelial structure (Fig. 6d, h, l and p). These observations uncovered the drastic structural re-organization of the midgut symbiotic organ in the fifth instar nymphs of *P. stali* as follows: (i) active constriction occurs in the most basal region of each crypt (ii); each crypt cavity is structurally isolated from the midgut main tract; (iii) consequently, the symbiotic bacteria are confined in each crypt cavity, which may hinder the symbiont movement to/from the midgut main tract; (iv) probably due to the basal constriction of the numerous crypts, the whole midgut symbiotic organ is structurally deformed, resulting in shortening, thickening and twisting in shape.

### Re-organization of antero-posterior midgut connection at late developmental stages of *P. stali*

**Fig. 7:**
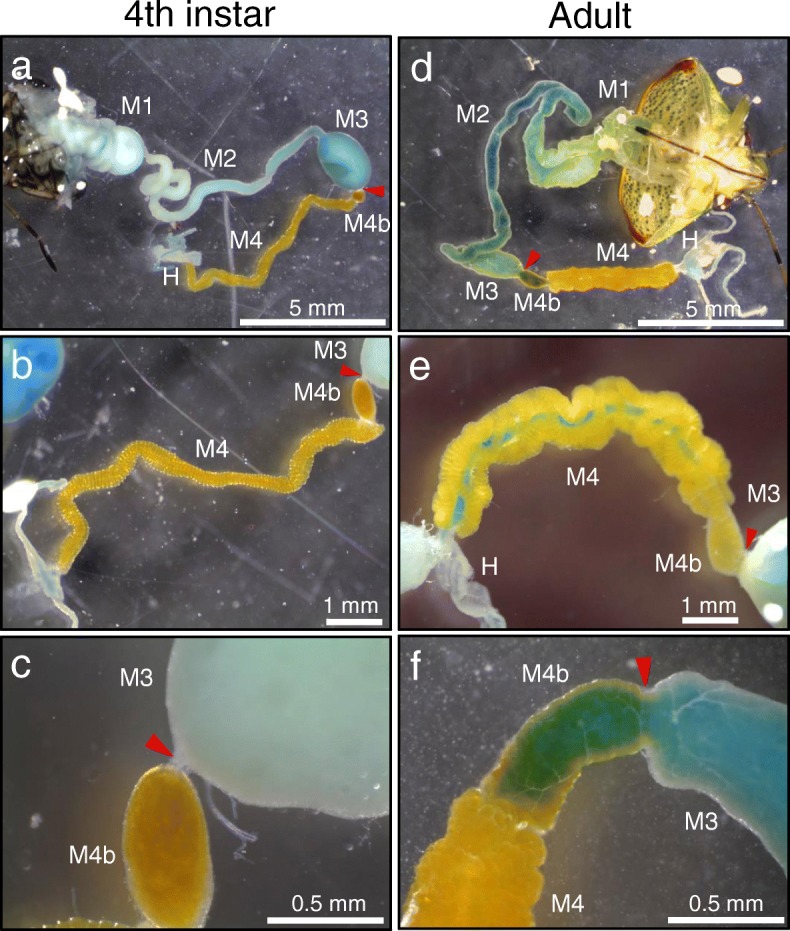
Alimentary tracts dissected from *P. stali* fed with colored water containing 0.05% Brilliant Blue FCF. **a–c** Fourth instar nymphs. **d–f** Adult insects. **a**, **d** Whole alimentary tract. **b**, **e** Around the symbiotic M4 region with crypts. Note that the main tract in the M4 region is not colored in the fourth instar nymph but deeply colored in the adult insect. **c**, **f** Around the M3–M4b junction. Red arrowheads indicate the constricted region that functionally separates the anterior and posterior regions of the midgut as a symbiont sorting organ that blocks food flow to the posterior midgut [44]. Abbreviations: M1, midgut first section; M2, midgut second section; M3, midgut third section; M4b, bulb-like region aboral to M4; M4, midgut fourth section with crypts; H, hindgut

Insects and other animals generally have a mouth for feeding, an anus for defecation, and a gut connecting them for digestion and absorption. However, Ohbayashi et al. [44] recently reported a striking discovery: in many stinkbug species, the mouth and the anus are structurally connected by a stretch of the alimentary tract, but functionally disconnected in the middle by a peculiar organ specialized for symbiont sorting. The tiny organ lies between the midgut M3 and M4b sections as a constricted region (red arrowheads in Fig. 7), which partitions the midgut into the anterior non-symbiotic region and the posterior symbiotic region. The organ blocks food fluid and non-symbiotic bacteria, but selectively allows passing of the specific bacterial symbiont, whereby the posterior symbiotic region is devoid of food flow, is free of non-symbiotic microbial contaminants, and maintains intimate and stable association with the specific bacterial symbiont. Ohbayashi et al. [44] mainly investigated all developmental stages of the bean bug, *Riptortus pedestris* (Alydidae), and also inspected adult insects of diverse stinkbugs (representing the families Coreidae, Rhyparochromidae, Pentatomidae, Scutelleridae, Dinidridae, Cydnidae, Acanthosomatidae and Plataspidae) that possess the midgut symbiotic organ, and observed the same phenomenon: when the insects were fed with water containing food colorings, the midgut M1, M2 and M3 regions were colored, but the midgut M4b and M4 regions were not colored. In the present study, when fourth instar nymphs of *P. stali* were fed with colored water and dissected, we observed that (i) the M3-M4b junction was strongly constricted, (ii) the midgut M1, M2 and M3 regions were colored, and (iii) no coloring was found in the midgut M4b and M4 regions (Fig. 7a-c), which were as previously reported in other stinkbug species [44]. By contrast, when adult insects of *P. stali* were fed with colored water and dissected, we found that (i) the M3-M4b junction was no longer constricted but broad in width, (ii) not only the midgut M1, M2 and M3 regions but also the midgut M4b and M4 regions were colored, (iii) in the midgut M4 region, the coloring was observed only in the main tract and not found within the crypts (Fig. 7d-f). The colored main tract of the M4 region was observed in both adult males and females. These observations suggested that the midgut symbiotic organ of *P. stali* exhibits not only structural but also functional re-organization during the fifth instar period.

## Discussion

**Fig. 8:**
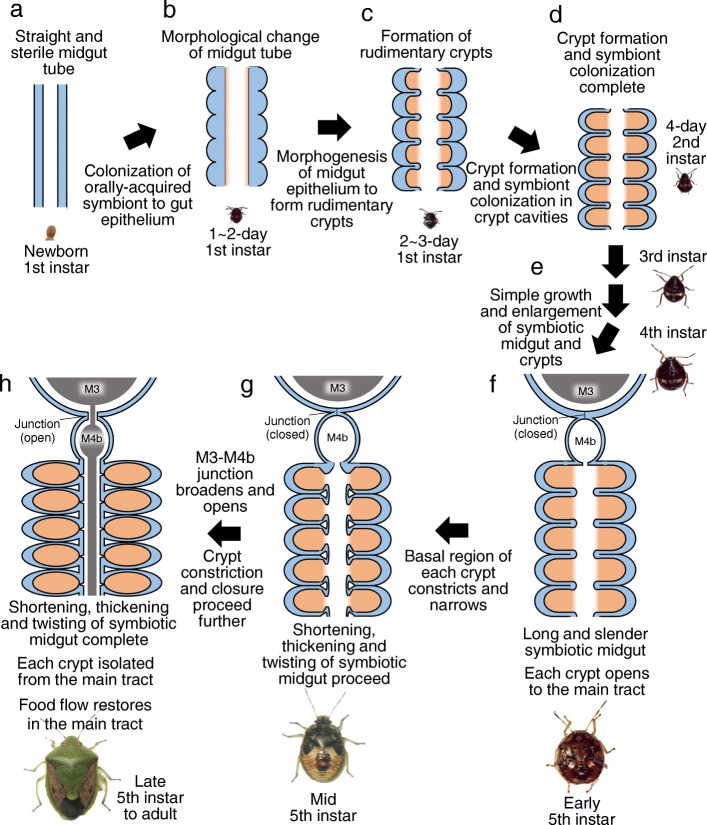
Schematic diagram of the morphogenetic events observed in the midgut symbiotic organ during the development of *P. stali*. Blue, orange, and gray show host’s intestinal epithelia, symbiotic bacteria, and food flow, respectively. **a** Newborn first instar nymph. **b** First instar nymph 1–2 days after hatching. **c** First instar nymph 2–3 days after hatching. **d** Second instar nymph on the day of molting. **e** Second, third and fourth instar nymphs. **f** Early fifth instar nymph. **g** Mid fifth instar nymph. **h** Late fifth instar nymph to adult. For detail, see the main text

In the present study, we provide the most detailed and comprehensive descriptions to date on the morphogenesis of the symbiotic organ and the process of symbiont colonization, using the gut symbiotic system of *P. stali* as an experimental model, which are schematically summarized in Fig. 8. Upon hatching, the newborn nymph, whose midgut is a simple and smooth tube, orally acquires the symbiotic bacteria from the egg surface (Fig. 8a). Subsequently, symbiont colonization to the posterior midgut occurs, and thickening and folding of the midgut epithelium proceed during the first instar period (Fig. 8b and c). By the second instar, rudimentary crypts are formed, and their inner cavities are colonized by the symbiotic bacteria (Fig. 8d). From the second to the fourth instar, while the alimentary tract grows and the posterior midgut is established as the symbiotic organ with numerous crypts, the anterior midgut and the posterior midgut are structurally and functionally isolated by a constricted region at the M3–M4b junction (Fig. 8e). By the early fifth instar, the midgut symbiotic organ attains the maximal length (Fig. 8f). Subsequently, toward the mid fifth instar, the basal region of each crypt starts to constrict and narrow, which deforms the midgut symbiotic organ as a whole into a shorter, thicker and twisted shape (Fig. 8g). By the late fifth instar to adulthood, the crypts are constricted off, by which the symbiotic bacteria are confined in the crypt cavities and isolated from the midgut main tract, and concurrently, the M3-M4b junction becomes thick and open, by which the food flow from the anterior midgut to the posterior midgut recovers (Fig. 8h).

We found that the alimentary tract of newborn nymphs of *P. stali* is initially a simple tube, but soon a series of morphogenetic events occur in their gut epithelium, and the rudimentary crypts are formed and colonized by the symbiotic bacteria by the second instar stage (Figs. 4 and 5). By performing RNA sequencing of the posterior midgut at this developmental stage, identifying specifically-expressed and/or up-regulated genes therein, and knocking-down these genes by RNA interference, we would be able to understand the molecular mechanisms underpinning the intricate morphogenesis of the midgut symbiotic organ, which entails formation of the numerous sac-like crypts arranged in four rows along the midgut main tract (see Fig. 1). Furthermore, by performing such analyses on symbiotic and aposymbiotic newborn nymphs in a comparative manner, we will be able to gain insight into how and to what extent the host and the symbiont respectively contribute to the morphogenetic processes of the midgut symbiotic organ.

We found that, during the period from the fifth instar to adulthood, despite the increasing body size, the midgut symbiotic organ becomes shorter, thicker and twisted (Fig. 4a). Close histological and cytological inspections revealed that the midgut symbiotic organ experiences characteristic structural re-organization during the fifth instar period, in which the basal region of each crypt is constricted off, each crypt cavity is isolated from the midgut main tract, and consequently the symbiotic bacteria are isolated and retained within the numerous crypt cavities along the midgut symbiotic region (Fig. 6). We propose the hypothesis that the tensile force collectively generated by the basal constriction of numerous crypts is responsible for the shortening, broadening and twisting of the midgut symbiotic organ during the fifth instar period. Plausibly, the crypt constriction may entail accumulation of cytoskeletal elements at the crypt bases, involvement of contractile machineries like actomyosin system and/or tubulin-dynein system, recruitment of programmed cell death or apoptosis, etc. In the future, we are planning to investigate these molecular and cellular processes by utilizing a variety of marker molecules, antibodies, RNA sequencing and RNA interference approaches. How these morphogenetic processes are affected by the symbiotic bacteria is of interest, but it is practically difficult to obtain aposymbiotic fifth instar nymphs of *P. stali* due to high mortality of aposymbiotic insects.

Strikingly, we found that, concurrent with the confinement of the symbiotic bacteria within the crypt cavities and consequent isolation from the midgut main tract during the fifth instar period, the constricted M3-M4b junction, which functionally separates the anterior midgut region for food digestion and absorption from the posterior midgut region for hosting the symbiotic bacteria, becomes thick and loose, thereby restoring the food flow across the anterior and posterior midgut regions (Fig. 7). By contrast, a previous detailed study on the bean bug *R. pedestris* revealed that the anterior midgut region is functionally isolated from the posterior midgut region without food flow at the M3-M4b junction throughout the nymphal and adult stages [44]. What factors are relevant to the difference between *R. pedestris* and *P. stali*?

A conceivable factor is the transmission mode of the symbiotic bacteria. Since *R. pedestris* acquires the *Burkholderia* symbiont from the environment at an early nymphal stage every generation [50, 51], the contaminant/symbiont ratio in the microbial inoculum ingested by the nymphs must be very high, which implies that exclusion of non-symbiotic microbe is very important for *R. pedestris*. On the other hand, adult females of *P. stali* smear an excrement containing the *Pantoea*-allied symbiont onto the eggshell upon oviposition [28, 47], and thus the symbiont inoculum ingested by the newborn nymphs from the eggshell contains the symbiotic bacteria of high purity, which may be relevant to the finding that the M3-M4b junction becomes less exclusive against foreign microbes in *P. stali* than in *R. pedestris*.

Another conceivable factor is the high reproductive activity of *P. stali*. Under a favorable rearing condition in the laboratory, adult females of *P. stali* start to lay eggs within 5 days after eclosion, and then continue to produce an egg mass consisting of about 14 eggs every 1 or 2 days for several weeks to months, which often amount to over 300–500 eggs in total (Oishi, unpublished data). In order to support the high level of reproductive activity in *P. stali*, a large amount of food must be efficiently ingested, digested and absorbed to acquire a sufficient quantity of nutrients by adult insects, which may be the reason why the food flow in the midgut of *P. stali* restores prior to the adult stage.

For many plant-sucking stinkbugs including *P. stali*, the gut symbiotic bacteria are essential for normal growth and survival [22–38], and thus stable vertical symbiont transmission is expected to be strongly selected for [14, 15, 52]. In this study, however, we observed that vertical symbiont transmission via maternal smearing onto the eggshell occasionally fails, which may result in symbiosis-deficient insects suffering growth defect and mortality (Fig. 3d; Fig. 5o). The incidence of the transmission failure may be an artifact under the laboratory rearing condition, or may be relevant to inbreeding of the host insect strain that had been maintained in the laboratory for years. Anyway, these observations highlight the importance of vertical symbiont transmission in stinkbugs.

In addition to *P. stali* (Pentatomidae) and *R. pedestris* (Alydidae), the functionally discontinuous midgut at the M3-M4b junction has been found in diverse plant-sucking stinkbugs representing the families Coreidae, Rhyparochromidae, Scutelleridae, Dinidridae, Cydnidae, Acanthosomatidae, and Plataspidae [44]. For understanding the diversity and evolution of the midgut symbiotic organ, similar morphological and histological studies on the development of the alimentary tract in diverse stinkbugs are anticipated, with particular emphasis on the morphogenetic, developmental, transcriptomic and functional aspects of the midgut crypts and the M3-M4b junction under a comparative perspective.

## Conclusions

In conclusion, we have described the morphogenesis and development of the midgut symbiotic organ and the process of symbiont colonization in an obligatory insect-bacterium gut symbiotic system in an unprecedented detail. Besides some old histological descriptions, there have been few comprehensive studies on the morphogenesis of the midgut symbiotic organ and the process of symbiont colonization in stinkbugs (reviewed in [16]). Considering that *P. stali* is recently emerging as a useful model system for experimentally studying the intimate insect-microbe gut symbiotic association [28, 47, 53], the knowledge obtained in this study is expected to establish the foundation for the further development of this research field.

## Data Availability

The raw data and the materials are available upon request to the authors.
